# Early characteristics of infants with pulmonary hypertension in a referral neonatal intensive care unit

**DOI:** 10.1186/s12887-017-0910-0

**Published:** 2017-07-11

**Authors:** Shilpa Vyas-Read, Usama Kanaan, Prabhu Shankar, Jane Stremming, Curtis Travers, David P. Carlton, Anne Fitzpatrick

**Affiliations:** 1 Pediatrics, Emory University School of Medicine, Atlanta, GA USA; 2 Sibley Heart Center, Pulmonary Hypertension Program, Atlanta, GA USA; 3 Department of Pediatrics, University of Colorado, Denver, CO USA; 4 Biostatistics, Pediatric Research Alliance, Atlanta, GA USA; 5 Division of Neonatology, Emory University School of Medicine, 2015 Uppergate Dr. NE, 3rd floor, 30322 Atlanta, GA USA

**Keywords:** Very low birth weight, Growth restriction, Caffeine, Pulmonary hypertension, Atrial septal defect

## Abstract

**Background:**

Approximately 8–23% of premature infants develop pulmonary hypertension (PH), and this diagnosis confers a higher possibility of mortality. As a result, professional societies recommend PH screening in premature infants. However, the risk factors for and the outcomes of PH may differ depending on the timing of its diagnosis, and little evidence is available to determine at-risk infants in the referral neonatal population. ﻿The objective of this study was to define clinical and echocardiographic characteristics of infants with pulmonary hypertension during the neonatal hospital course and at or near-term﻿.

**Methods:**

Infants who had the following billing codes: < 32 weeks, birth weight < 1500 g, neonatal unit, and echocardiograph had records abstracted from a data warehouse at Children’s Healthcare of Atlanta. The outcome was defined as late PH on the final echocardiogram for all patients, and, separately, for patients with multiple studies. Descriptive statistics, univariable, and multivariable models were evaluated, and odds ratios and 95% confidence intervals are expressed below as (OR, CI).

**Results:**

556 infants were included in the overall study, 59 had PH on their final echocardiogram (11%). In multivariable analyses, atrial septal defect (2.9, 1.4–6.1), and intrauterine growth restriction (2.7, 1.2–6.3) increased the odds of late PH, whereas caffeine therapy decreased PH (0.4, 0.2–0.8). When the analyses were restricted to 32 infants who had multiple echocardiograms during their hospitalization, the association between atrial septal defect (5.9, 2.0–16.5) and growth restriction (3.7, 1.3–10.7) and late PH was strengthened, but the effect of caffeine therapy was no longer significant. In this smaller subgroup, infants with late PH had their final echocardiogram at a median of 116 days of life, and 42–74% of them had right ventricular pathology.

**Conclusions:**

Early clinical variables are associated with PH persistence in a referral neonatal population. Identification of early clinical factors may help guide the ascertainment of infant risk for late PH, and may aid in targeting sub-groups that are most likely to benefit from PH screening.

## Background

For infants who are born early in the third trimester of pregnancy or before, fetal factors coupled with prematurity and postnatal injury may combine to result in pulmonary vascular disease, a process which manifests clinically as pulmonary hypertension (PH) [1–4]. The incidence of PH in very low birth weight infants is estimated to range between 16 and 43% [5–8], and infants with bronchopulmonary dysplasia and PH have been suggested to have up to a 4.6-fold higher odds of mortality than those infants with bronchopulmonary dysplasia without PH [5–7, 9]. Further, infants who were born prematurely continue to have an increased risk for PH that persists into childhood and adolescence [10].

Given the potential for neonatal and post-neonatal mortality, professional societies’ such as the American Heart Association and the American Thoracic Society recommend screening premature infants for the development of PH [11]. Unfortunately, few predictors for the development of PH are known. The most widely studied predictor for PH is bronchopulmonary dysplasia (BPD). Among infants with the most severe form of BPD, 29 to 53% also had a diagnosis of PH, and infants with severe BPD have over a 6-fold increased odds of PH when compared to infants with less severe lung disease [1, 12–15]. Intrauterine growth restriction has also been associated with PH, suggesting that fetal vascular programming may play a role in the development of hypertension [5, 16–18]. Few other potential predictors have been identified other than these, postnatal infection, and possibly illicit drugs [14, 19, 20].

In this study, we utilized observational data from very low birth weight, premature infants within our referral population to determine if early clinical factors are associated with pulmonary hypertension. To our knowledge, this cohort is the largest sample of neonates who received echocardiograms to be studied for PH. Because factors that contribute to the development of PH, such as infant growth and mortality, may differ in Identification of early clinical factors that are associated with neonatal PH may aid in identifying an “at risk” population that may require more echocardiographic surveillance during the hospital stay or more formal follow-up after hospital discharge.

## Methods

### Study design

This study was approved by the Institutional Review Board at Emory University and at Children’s Healthcare of Atlanta. The study population was a retrospective, observational cohort of patients at two hospitals in Atlanta (Children’s Healthcare of Atlanta, Egleston or Scottish Rite campus) from January 2010 to September 2014. Infants who were less than 32 weeks gestational age at birth, had a birth weight of less than 1500 g, were in the neonatal intensive care unit, and had an echocardiogram were identified using ICD-9 codes and were included. Patients were excluded if medical records were missing or if they had multiple anomalies/aneuploidy, congenital heart disease (other than atrial septal defect, ventricular septal defect, or patent ductus arteriosus), or congenital lung disease.

### Definitions of exposures and outcomes

#### Outcomes

The primary outcome was late pulmonary hypertension (late PH) on the final echocardiograph prior to death or discharge from the neonatal intensive care unit. Pulmonary hypertension (PH) was defined as an echocardiogram that showed: 1) a moderate-to-large patent ductus arteriosus (PDA) with bidirectional or right-to-left shunting; 2) a tricuspid regurgitation jet gradient of ≥32 mmHg with septal flattening, right ventricular hypertrophy, or right ventricular dilation; or 3) a tricuspid regurgitation jet velocity of ≥45 mmHg [16, 19, 21]. Echocardiographs were ordered at the discretion of the attending neonatologist and interpreted by pediatric cardiologists.

#### Clinical variables

The following variables were abstracted from the infant medical record: 1) maternal drug use, the use of tobacco and/or alcohol; 2) illicit drug use, including the use of illegal drugs such as cannabis, amphetamines, or other substances; 3) infant race and gender; and 4) prenatal and intrapartum complications. The following discrete variables were abstracted from the clinical data warehouse using ICD-9 codes: 1) intraventricular hemorrhage; 2) necrotizing enterocolitis; 3) retinopathy of prematurity; 4) medication use; 5) respiratory support; and 6) positive blood culture. Death was defined as mortality from any cause during the hospital course.

#### Echocardiographic variables

The echocardiographic characteristics of infants with late PH were determined by the final echocardiogram of the hospital stay. For infants with only one study, the final echocardiogram was the infant’s first study in our neonatal hospital system. For infants with more than one study, the final echocardiogram was the infant’s last study in our neonatal hospital system. Directionality of the shunt through an ASD, ventricular septal defect, or patent ductus arteriosus (PDA) was determined by the pediatric cardiologist at the time of the echocardiogram as 1) left-to-right or none 2) bidirectional or 3) right-to-left. The tricuspid regurgitation jet velocity (TRJV) was graded as 1) normal, < 32 mmHg 2) mild, 32–44 mmHg 3) moderate 45–60 mmHg and 4) severe ≥60 mmHg at that time. Septal flattening was defined subjectively as none, any, or severe by the pediatric cardiologist performing the echocardiogram. Right ventricular dilatation, hypertrophy, and dysfunction were defined as either present or absent. Atrial septal defects were categorized as 1) none or patent foramen ovale (PFO), 2) patent foramen ovale versus atrial septal defect (PFO vs. ASD), or 3) atrial septal defect (ASD). PDA was defined as 1) none or small or ligated versus 2) moderate-to-large on the first study echocardiogram. Ventricular septal defects were defined as 1) intact, tiny, and small or 2) moderate-to-large or multiple.

#### Descriptive statistics

Two-sample t-tests for normally distributed variables, and Wilcoxon rank sum tests for skewed distributions were utilized. For categorical variables, chi-square tests of proportion were used to compare outcome groups unless the cell frequency was ≤5, in which case the Fisher’s exact test was used.

#### Univariable and multivariable analyses

To determine the effect of echocardiographic and early hospital characteristics on the outcome of late PH, univariable logistic regression was used to arrive at odds ratios, and 95% confidence intervals.

A multivariable model was constructed by the manual addition of each significant variable to the intercept and gestational age variable. The -2Log likelihood values were determined, and the value of each additional variable was determined using a likelihood ratio test with *p* ≥ 0.05 as a stopping rule. A final model that included gestational age, atrial septal defect (ASD vs. None or PFO), intrauterine growth restriction (yes or no), caffeine (yes or no), and positive-pressure ventilation at 28 days (yes vs. no) was derived. Although gestational age and positive-pressure ventilation were not statistically significantly different between groups, they were forced into the model as important risk factors for the development of late PH. Additionally, the defined multivariable model was evaluated again in infants who had more than one echocardiogram performed, and who had evidence of late PH on this final study. Odds ratios and 95% confidence intervals were constructed. All statistical procedures were performed using SAS 9.4 statistical software and the level of significance for comparisons was a *p*-value <0.05.

## Results

### Patient selection

**Fig. 1:**
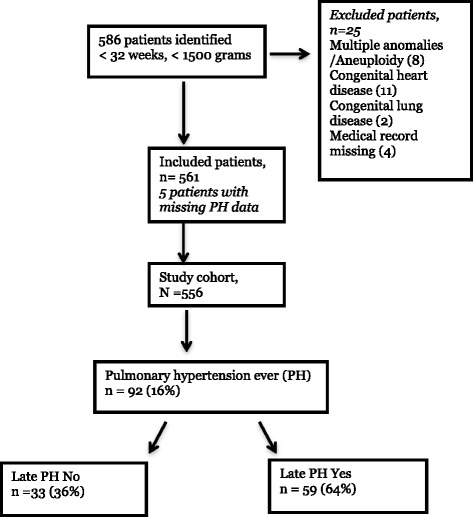
Flowchart of study patient selection. An electronic health record database query was performed for <32 weeks gestation at birth, < 1500 g birthweight, neonatal intensive care unit, and echocardiographic procedure. 586 infants were identified, and 25 of these met exclusion criteria. 556 patients were included in the study, and 92 (16%) had PH on any one echocardiogram while in the neonatal intensive care unit. Of the 92 infants with PH, 59 (64%) also had PH on their final echocardiogram of the hospital stay. Of these 59 infants, 32 had more than one echocardiogram and the last study during the neonatal stay showed PH

**Fig. 2:**
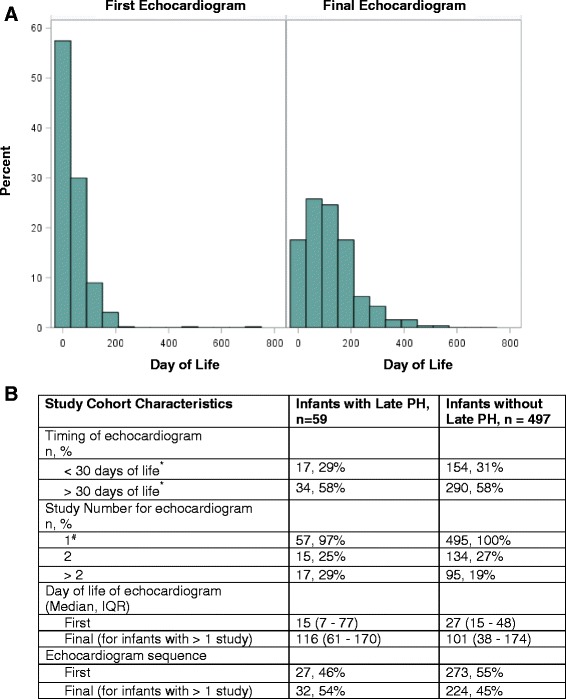
Description of the timing and number of echocardiograms in infants with and without late PH. Infants were categorized as having late pulmonary hypertension (late PH) if their final echocardiogram in the hospital showed PH. For infants with only one echocardiogram, the final study was their first echocardiogram. For infants with more than one study, the final echocardiogram of the neonatal course was captured. Echocardiograms were performed by clinical pediatric cardiologists and quantitative variables (tricuspid regurgitation jet velocity) and qualitative variables (shunt directions, septal flattening, degree of right ventricular dysfunction/dilation/hypertrophy) were measured. Panel **a** shows the distribution of the day of life for the first and final echocardiogram of the infants in the entire cohort. Panel **b** shows the day of life that the echocardiograms were obtained, the number of studies by group, and the day of life and sequence of the first and final echocardiogram. * missing day of life information in the late PH group (*n* = 8), and in the without late PH group (*n* = 53). ^#^2 infants had missing study number information in both groups

An initial search of the electronic health record (Clarity) database from January 2010 to January 2015 yielded 586 infants that met the inclusion criteria of having a gestational age of less than 32 weeks at birth, having a birthweight less than 1500 g at birth, being admitted to the neonatal intensive care unit, and having an echocardiographic procedure (Fig. 1). Of these, 25 infants were excluded from the study population due to multiple anomalies or aneuploidy (8 infants), congenital heart disease other than PDA, ASD or VSD (11 infants), congenital lung disease (2 infants), or missing medical information (4 infants). Five patients were excluded due to missing outcome data, and 556 infants were included in the study cohort. Ninety-two infants (16%) in the overall cohort had at least one echocardiogram that met criteria for pulmonary hypertension (PH) during the hospital course. Infants who had echocardiographic evidence of pulmonary hypertension on their last neonatal study were defined as having late pulmonary hypertension (late PH). If an infant had only one study, then the first study determined the infant’s classification into outcome groups. If an infant had more than one study, then the final echocardiogram result determined whether he/she was classified as having late PH. The majority of infants with and without late PH had an echocardiogram after 30 days of life, and 29–31% of infants had early echocardiograms before 30 days of life (Fig. 2). The median day of life for infants with over one study was 116 days for infants with late PH, and 101 for infants without late PH, indicating that PH on the final echocardiogram was detected beyond 3 months of life for this group (Fig. 2, panel B). All infants in both groups received at least one echocardiogram. Twenty-five percent of infants with late PH, and 27% of infants without late PH also had a second echocardiogram. A higher proportion of infants in the late PH group had over 2 echocardiograms when compared with infants without late PH (29% vs. 19%). Forty-six percent of infants in the late PH group had evidence of PH on their first and only echocardiogram, and 54% of infants had over one study and the final echocardiogram showed PH.

### Echocardiographic characteristics of infants with late PH

Of the 556 infants evaluated, 59 patients had late PH on their final echocardiogram (10.6% of the total cohort, 64% of the infants with an initial diagnosis of PH), and 497 infants did not (Table 1). The median timing of the echocardiogram was 77 days of life for infants in the late PH group, and 41 days of life for infants without late PH. Infants who had an atrial septal defect (ASD) had 4.6-fold higher odds of late PH, when compared with infants without an ASD. The association between patent ductus arteriosus size and late PH was not significant. Forty-three percent of infants with late PH had an ASD with bidirectional or right-to-left shunting, indicating that patients in our PH group had poor right ventricular compliance likely due to the effect of chronically elevated pulmonary pressure. Eighty-one percent of infants with late PH had tricuspid jet velocities of ≥32 mmHg, compared with 2% of patients without late PH. The 11 infants with “normal” tricuspid regurgitation in the late PH group also had a patent ductus arteriosus, making interpretation of the jet gradient difficult. The majority of infants with late PH had some evidence of either right ventricular dilation (55% vs. 8%) or right ventricular hypertrophy (56% vs. 10%), and one-third had right-ventricular dysfunction (33% vs 3%), respectively, when compared with those without late PH. Ventricular septal defects and left ventricular dysfunction did not differ between the late PH groups.

**Table 1:** Echocardiographic characteristics of infants with late pulmonary hypertension

Echocardiographic parameters	Infants with Late PH, *n* = 59	Infants without Late PH, *n* = 497	Odd ratio (95% CI)	*p* - value
Timing of final echo Median (IQR)	77 (13–136)	41 (21–105)	1.0 (1.0–1.0)	0.39
Atrial septal defects				
None/PFO	43, 73%	434, 87%	ref	
PFO vs. ASD	4, 7%	24, 5%	1.7 (0.6–5.1)	0.68
ASD	10, 17%	22, 4%	4.6 (2.0–10.3)	<0.01*
Atrial shunt direction (n, %)				
None or left-to-right	29, 57%	312, 87%	ref	
Bidirectional/Right-to-Left	22, 43%	47, 13%	5.0 (2.7–9.5)	<0.01*
Patent Ductus Arteriosus				
None	16, 36%	217, 54%	ref	
Small	11, 24%	69, 17%	2.2 (1.0–4.9)	0.28
Moderate/Large	18, 40%	116, 29%	2.1 (1.0–4.3)	0.27
Patent ductus arteriosus shunt direction (n, %)				
None or left-to-right	12, 43%	177, 99%	ref	
Bidirectional/Right-to-Left	16, 57%	1, < 1%	236.0 (29–1933)	<0.01*
Tricuspid regurgitation jet velocity (n, %)				
Normal, < 32 mmHg	11, 19%	474, 98%	ref	
Mild, 32–44 mmHg	23, 39%	12, 2%	83.0 (33–207)	< 0.01*
Mod, 45–60 mmHg	15, 25%	0, 0%	________	____
Severe, > 60 mmHg	10, 17%	0, 0%	________	____
Septal flattening (n, %)				
None	1, 2%	96, 43%	ref	
Any	48, 98%	126, 57%	36.6 (5.0–269.7)	< 0.01*
Right ventricular dilation (n, %)				
None	25, 45%	436, 92%	ref	
Any	31, 55%	36, 8%	15.0 (8.0–28.1)	< 0.01*
Right ventricular hypertrophy (n, %)				
None	22, 44%	427, 90%	ref	
Any	28, 56%	50, 10%	10.9 (5.8–20.4)	< 0.01*
Right ventricular dysfunction (n, %)				
None	38, 67%	472, 97%	ref	
Any	19, 33%	14, 3%	16.9 (7.8–36.2)	< 0.01*
Ventricular Septal Defect				
None/Small	51, 96%	421, 97%	ref	
Mod/Large	2, 4%	11, 3%	1.5 (0.3–6.9)	0.60
Left ventricular dysfunction (n, %)	7, 12%	31, 6%	2 (0.8–4.8)	0.11

Infants were categorized as having late pulmonary hypertension (late PH) if their final echocardiogram in the hospital showed PH. Echocardiograms were performed by clinical pediatric cardiologists and quantitative variables (tricuspid regurgitation jet velocity) and qualitative variables (shunt directions, septal flattening, degree of right ventricular dysfunction/dilation/hypertrophy) were measured. Odds ratios were defined using univariable logistic regression, *p < 0.05 is significant

### Associations between neonatal characteristics and late PH

In univariable analysis, infants with late PH overall had lower birthweight than infants who did not have PH persistence (0.75 ± 0.24 kg vs. 0.83 ± 0.25 kg, *p* = 0.02) (Table 2). For each kilogram increase in an infant’s birthweight, the odds of PH dropped significantly (OR 0.2, 95% CI 0.1–0.7), and infants with intrauterine growth restriction had higher odds of developing late PH than normally grown infants (OR 2.9; 95% CI 1.3–6.2). Interestingly, an association between caffeine and late PH was also seen. Fifty-one percent of infants with late PH received caffeine, compared with 69% of infants without late PH (OR 0.5; 95% CI 0.3–0.8). Despite this discrepancy in caffeine use, we did not detect a difference in the level of respiratory support at 28 days of life or at 36 weeks corrected gestational age between groups. Nineteen percent of infants with late PH were treated with sildenafil, and 3% also were treated with bosentan. Other birth variables and early hospital characteristics did not differ between the comparison groups.

**Table 2:** Evaluation of birth and early hospital characteristics of infants with late PH

Birth/early hospital characteristics	Infants with Late PH, *n* = 59	Infants without Late PH, *n* = 497	Odds ratio (95% CI)	*p* - value
Apgar 1 min. Mean ± SD	3.5 ± 2.8	4.1 ± 2.6	0.9 (0.8–1.0)	0.10
Apgar 5 min. Mean ± SD	6.3 ± 2.4	6.5 ± 2.2	1.0 (0.8–1.1)	0.44
Birthweight (kg) Mean ± SD	0.75 ± 0.24	0.83 ± 0.25	0.2 (0.1–0.7)	0.02*
Gestational age (wks) Mean ± SD	26.1 ± 2.4	26.1 ± 2.2	1.0 (0.9–1.1)	0.99
Mode of Delivery (n, %)				
C/S	44, 77%	327, 66%	1.7 (0.9–3.3)	0.10
Vaginal	13, 23%	166, 34%	ref	
Gender (n, %)				
Male	33, 56%	289, 42%	0.9 (0.5–1.6)	0.71
Female	26, 44%	205, 42%	ref	
Intrauterine growth restriction (n, %)	10, 18%	35, 7%	2.9 (1.3–6.2)	< 0.01*
Placental abruption (n, %)	9, 16%	49, 10%	1.7 (0.8–3.7)	0.17
Chorioamnionitis (n, %)	3, 5%	50, 10%	0.5 (0.2–1.7)	0.27
Maternal betamethasone (n, %)				
2 or more doses	27, 57%	267, 62%	0.8 (0.4–1.5)	0.52
0–1 doses	20, 43%	162, 38%	ref	
Race (n, %)				
Black	36, 61%	290, 60%	1.1 (0.6–1.8)	0.84
White/Asian/Hispanic	23, 39%	196, 40%	ref	
Illicit drug use (n, %)	3, 6%	41, 9%	0.6 (0.2–2.2)	0.47
Multiples (n, %)	12, 21%	91, 18%	1.2 (0.6–2.3)	0.68
Maternal drug use	5, 10%	39, 9%	1.2 (0.4–3.1)	0.76
Caffeine (n, %)	29, 51%	341, 69%	0.5 (0.3–0.8)	< 0.01*
Any respiratory support, 28 days (n, %)	26, 44%	212, 43%	1.0 (0.6–1.8)	0.87
Positive pressure ventilation, 28 days (n,%)	26, 44%	203, 41%	1.1 (0.7–2.0)	0.63
Any respiratory support, 36 weeks (n, %)	26, 44%	199, 40%	1.2 (0.7–2.2)	0.45
Sildenafil (n, %)	11, 19%	19, 4%	5.8 (2.6–12.8)	< 0.01*
Bosentan	2, 3%	0, 0%	__________	_____

The birth and early hospital characteristics of infants who had late PH were compared to those infants who did not have late PH using univariable logistic regression. Odds ratios were defined using univariable logistic regression, *p < 0.05 is significant

Because outcomes and predictors may differ based on the timing of PH diagnosis, we then evaluated early clinical and echocardiographic factors in infants who had multiple studies and had an echocardiogram showing PH on their final study [21]. This subgroup of 32 infants had their final echocardiogram between 101 days (infants without late PH) and 116 days (infants with late PH) (Table 3). The gestational age of infants with multiple studies ranged from 25.7 to 25.9 weeks in both groups, placing the final echocardiogram between 39 and 41 weeks corrected gestational age. Infants in this subgroup with late PH had an Apgar score that was significantly lower than their counterparts who did not have late PH (3.0 vs. 4.3, *p* = 0.01). More than double the infants with multiple studies and late PH had a history of intrauterine growth restriction, when compared with infants without late PH (23% vs. 9%, *p* = 0.03). In this subgroup that was evaluated later in the neonatal course, caffeine therapy was not associated with the outcome of late PH (63% vs. 68%, *p* = 0.55). Interestingly, the use of respiratory support at 28 days or 36 weeks again did not differ between groups. Four times the proportion of infants with multiple studies and late PH had atrial septal defects, when compared with those without late PH (28% vs. 7%, *p* < 0.001). Although death rates during the neonatal hospitalization were similar in late PH groups, the proportion of infants with bidirectional or right-to-left shunts through an ASD or PDA was much higher in infants who had late PH on their final echocardiogram (46% vs. 12% and 57% vs. 0%, respectively). Further, a large proportion of infants with multiple studies and late PH had some degree of septal flattening (97% vs. 53%, *p* < 0.001), right ventricular dilation (69% vs. 11%, *p* < 0.001), right ventricular hypertrophy (74% vs. 17%, *p* < 0.001), or right ventricular dysfunction (42% vs. 5%, *p* < 0.001) on their final echocardiogram, indicating this that this subgroup was a very high-risk and high-acuity group within a sick, referral neonatal population.

**Table 3:** Comparison of characteristics between infants who had multiple studies by late PH status

Birth/early hospital characteristics	Infants with multiple studies and late PH *n* = 32	Infants with multiple studies without late PH *n* = 224	*p* - value
Apgar 1 min. Mean ± SD	3.0 ± 2.4	4.3 ± 2.5	0.01*
Apgar 5 min. Mean ± SD	6.0 ± 2.3	6.5 ± 2.2	0.27
Birthweight (kg) Mean ± SD	0.68 ± 0.19	0.80 ± 0.24	<0.01*
Gestational age (wks) Mean ± SD	25.7 ± 2.3	25.9 ± 2.2	0.73
Mode of Delivery – C/S (n, %)	23, 72%	145, 65%	0.44
Gender -- Male (n, %)	16, 50%	129, 58%	0.42
Intrauterine growth restriction (n, %)^#^	7, 23%	20, 9%	0.03*
Placental abruption (n, %)^#^	4, 13%	24, 11%	0.76
Chorioamnionitis (n, %)^#^	2, 6%	22, 10%	0.75
Maternal betamethasone (n, %)	16, 59%	122, 63%	0.72
Multiples (n, %)^#^	3, 9%	46, 21%	0.16
Caffeine (n, %)	20, 63%	152, 68%	0.55
Any respiratory support, 28 days (n, %)	17, 53%	113, 51%	0.81
Any respiratory support, 36 weeks (n, %)	21, 66%	117, 52%	0.35
Death (n, %)	12, 20%	86, 17%	0.56
Day of life, death or discharge Mean ± SD	113 ± 81	126 ± 74	0.61
Race (n, %)			0.52
Black	21, 66%	132, 60%	
Other	11, 34%	89, 40%	
Atrial septal defects (n, %)	9, 28%	16, 7%	<0.001*
Bidirectional/right-to-left atrial shunt (n, %)	12, 46%	18, 12%	<0.001*
Patent Ductus Arteriosus (n, %)			
Moderate/Large^#^	4, 19%	22, 14%	0.51
Bidirectional/R-to-L^#^	4, 57%	0, 0%	< 0.001*
Septal flattening (n, %)	29, 97%	79, 53%	<0.001*
Right ventricular dilation (n, %)	20, 69%	23, 11%	<0.001*
Right ventricular hypertrophy (n, %)	20, 74%	36, 17%	<0.001*
Right ventricular dysfunction (n, %)	13, 42%	11, 5%	<0.001*
Ventricular Septal Defect – Mod/Lgt^#^ (n, %)	1, 4%	2, 1%	0.35
Left ventricular dysfunction (n, %)^#^	3, 9%	16, 7%	0.67

For infants who had more than one study in the neonatal intensive care unit, clinical characteristics were compared between late PH groups. Two-sample t-tests or Wilcoxon rank sum test for continuous variables, or Chi-square/Fisher’s exact test for categorical variables were used. *p < 0.05 for comparison groups. ^#^Fisher’s Exact Test used

### Multivariable model for the outcome of late PH

To assess how each co-variate found to be significant in our univariable model affected the outcome of late PH when other early characteristics were adjusted for, we generated a multivariable model that evaluated gestational age, atrial septal defect, intrauterine growth restriction, caffeine therapy, and positive-pressure ventilation at 28 days on late PH (Table 4, A). Gestational age, instead of birth weight, was utilized as a co-variate because of potential collinearity between birth weight and intrauterine growth restriction. When other factors were controlled, infants with ASD had a 2.9-fold higher odds of late PH, compared to infants with no significant atrial shunts (95% CI 1.4–6.1). In this cohort, gestational age did not significantly increase the odds of late PH. However, infants with intrauterine growth restriction continued to have a 2.7-fold higher odds for late PH, even when other significant variables were controlled (95% CI 1.2–6.3). Infants receiving caffeine therapy had a lower probability of late PH than those who did not receive caffeine (OR 0.4; 95% CI 0.2–0.8). Positive-pressure ventilation was not significantly associated with late PH, when other factors were controlled (OR 1.5, 95% CI 0.8–2.9).

**Table 4:** Multivariable model for the outcome of late PH in all infants and in infants with multiple studies

Variable	Odds ratio	95% CI	*p*-value
Overall multivariable model for the outcome of late PH			
Gestational age	0.9	0.8–1.1	0.22
Atrial septal defect (ASD vs. None)	2.9	1.4–6.1	<0.01*
Growth restriction	2.7	1.2–6.3	0.02*
Caffeine	0.4	0.2–0.8	0.01*
Positive-pressure ventilation	1.5	0.8–2.9	0.16
Multivariable model for the outcome of late PH, among infants with multiple studies			
Gestational age	0.9	0.7–1.1	0.18
Atrial septal defect (ASD vs. None)	5.9	2.0–16.5	<0.001*
Growth restriction	3.7	1.3–10.7	0.01*
Caffeine	0.8	0.3–1.8	0.53
Positive-pressure ventilation	1.4	0.6–3.3	0.38

A) The association between significant co-variates and the outcome of late pulmonary hypertension was evaluated. B) The association between significant co-variates and the outcome of late pulmonary hypertension in infants with multiple studies was evaluated. Gestational age and positive-pressure ventilation were forced into the model. Multivariable logistic regression was used to derive odds ratios and 95% confidence intervals. *p ≤ 0.05 was significant

When the model was evaluated in the sicker subgroup of infants who had multiple studies, infants with an ASD had even higher odds of late PH than those who did not have significant ASD, with other variables being held constant (OR 5.9, 95% CI 2.0–16.5). Similarly, infants with multiple studies who had growth restriction had a 3.7-fold increase in the odds of late PH, when compared with normally grown infants (95% CI 1.3–10.7). Interestingly, the effect of caffeine on late PH was no longer significant in this small subgroup of infants, when gestational age, ASD, growth restriction and positive-pressure ventilation were controlled (OR 0.8, 0.3–1.8).

## Discussion

In this study, we identify important risk factors for PH in a referral neonatal population including birthweight, the presence of an atrial septal defect, intrauterine growth restriction, and caffeine therapy. Further, we have shown that atrial septal defect and intrauterine growth restriction are significantly associated with late PH in infants who are at or near-term gestation.

In our multivariable model, the presence of an atrial septal defect increased the odds of late PH significantly, even when gestational age, growth restriction, caffeine use, and positive-pressure ventilation were controlled. Further, this association was strengthened in a subgroup of infants who had their final echocardiogram showing PH at or near term. Supporting these findings, other investigators have also found an association between the presence of an ASD and the development of PH in infants with BPD, a diagnosis that conferred an increased risk of mortality [22, 23]. Animal models of chronic left-to-right shunting have increased pulmonary vascular resistance and arteriolar medial thickness, implying biologic plausibility to the association between ASD and PH [24, 25]. Epidemiologically, ASDs account for 8–10% of congenital heart defects, though the majority of these close spontaneously in the first year of life [26]. For those with persistent defects, small studies have suggested that infants with BPD may have improved respiratory outcome following transcatheter closure of left-to-right shunts at approximately 6 months of age [27, 28]. Unfortunately, our finding that ASDs were associated with PH development is difficult to interpret causally. ASDs may cause right heart enlargement and diastolic septal flattening due to volume load, which may be confused with systolic septal flattening due to PH. In a similar manner, the presence of a large PDA may complicate the diagnosis of PH by echocardiography by elevating the right ventricular pressure due to transmission of aortic pressure to the pulmonary artery in the absence of elevated pulmonary resistance. However, 43–57% of infants in our analyses had bidirectional or right-to-left shunts, offering some reassurance that our findings are the result of elevated pulmonary resistance and not echocardiographic detection bias due to left-to-right shunting. Further, over half of the infants with late PH also had either right ventricular dilation or hypertrophy, supporting our hypotheses that these infants suffered from chronically elevated pulmonary resistance.

Intrauterine growth restriction was associated with pulmonary hypertension in descriptive analyses in our investigation, and it continued to be associated with PH when other clinical factors were controlled. Infants who had intrauterine growth restriction had a 2.9-fold higher odds of late PH when compared to normally grown infants, and this association was enhanced in our subgroup of infants who had late PH near term (OR 5.9 for late PH). In a single center study by Check et al., infants with BPD, who had a birth weight for gestational-age ratio percentile of less than 25%, had a 3.9-fold increase in the odds of PH at 36 weeks’ gestation. When this model was further controlled for gestational age, multiple gestation, gender and race, the odds of PH for growth-restricted infants were increased to 5.9, similar to our findings [16]. Our population differed slightly from that in the prior study, in that we did not restrict our cohort only to infants with BPD, and the overall proportion of infants with intrauterine growth restriction was only 8% (compared with 30%). Because evidence suggests that fetal factors potentially influence the propensity for disease in childhood and adulthood, the association between intrauterine growth restriction and PH should likely be interrogated further [29–31].

Interestingly, caffeine therapy strongly decreased the odds of late PH in our study overall. When gestational age, ASD status, growth restriction, and positive-pressure ventilation were controlled, the potentially protective effect of caffeine therapy on late PH was significant (OR 0.4, 95% CI 0.2–0.8). However, when the cohort was restricted to infants who had late PH later in life (the multiple studies group), the effect of caffeine on the outcome was no longer significant (OR 0.8, 95% CI 0.3–1.8). This differential effect in the association between caffeine therapy and the outcome of late PH may be secondary to the smaller numbers and potentially inadequate power in our subgroup analysis. Alternatively, it is possible that the effect of caffeine therapy on PH during the neonatal hospitalization is mediated through bronchopulmonary dysplasia. In support of this possibility, others have shown that caffeine therapy, particularly when initiated early in life, is associated with a reduction in bronchopulmonary dysplasia and ventilation time for premature neonates [32–34]. Additionally, it is possible that infants who are corrected to near-term and continue to have late PH are a different population than their younger counterparts, and the neonatal predictors of pulmonary hypertension at differing points during the postnatal hospital stay should continue to be carefully characterized.

We acknowledge that pulmonary hypertension based on echocardiographic parameters is challenging to define due to difficulty in obtaining a measurable tricuspid regurgitation jet velocity, and poor sensitivity and specificity for the detection of PH [35]. However, echocardiography is widely used in neonatal intensive care units because safety considerations prohibit the use of cardiac catheterization as a screening tool and clinical decisions are often made based on echocardiographic findings [36]. Further, the definitions of PH applied in our study are comparable to those used in other investigations, and studies have shown strong agreement between clinical and research echocardiograms for the detection of PH [7, 8, 12, 37]. In our cohort, infants were defined as having PH if they had a bidirectional or right-to-left patent ductus arteriosus shunt (indicating systemic or supra-systemic pulmonary vascular resistance), or a mildly elevated tricuspid regurgitation jet velocity with septal flattening, right ventricular hypertrophy or dilation, or a tricuspid regurgitation jet velocity of over 45 mmHg. Although a discrete number for the tricuspid regurgitation jet velocity may be difficult to obtain, we were able to group infants into having a tricuspid regurgitation jet that was “normal” (< 32 mmHg) or “abnormal” (> 32 mmHg). In other investigations, a measurable tricuspid jet velocity in combination with septal flattening, right ventricular hypertrophy, or right ventricular dilation had a positive predictive value of 89–100% [35]. Given these values, our patients who were classified as having PH echocardiographically would also be likely to have PH if they underwent catheterization. Longer-term follow-up and serial echocardiography may be useful to increase the positive prediction of echocardiogram for the development of PH. Additionally, although the optimal echocardiographic parameters for infants at risk of PH is not known, algorithms to detect the disease in a systematic manner are now being developed based on available literature [36].

The strengths of our study include its evaluation of a referral population of high-acuity neonates at particular risk for PH. To our knowledge, this is the largest sample of premature neonates who have received echocardiograms to be studied for pulmonary hypertension. Additionally, we included objective clinical variables in our models that would be readily available early in the neonatal hospital course, and steered away from variables that have traditionally been associated with PH but are present later in the neonatal course. We focused on utilizing these variables to begin to assess if early clinical factors can be utilized to determine infant risk for PH. Further, we have found strong associations between ASD and late PH and growth restriction and late PH, raising the question of whether premature infants with these particular risk factors should be evaluated earlier or more frequently for pulmonary hypertension, than those without these risks.

In summary, in this study, we have confirmed clinical variables that were known to be associated with the development of PH in premature infants, such as intrauterine growth restriction, and we have identified new factors that are associated with PH persistence, such as caffeine use and atrial septal defect. Our multivariable model for persistence of PH utilizes only readily available clinical variables, rather than solely relying on echocardiographic or respiratory characteristics, to establish risk for PH. Echocardiographic and respiratory variables may vary by clinician judgement, and utilizing these variables for prediction of PH may prove to be challenging and difficult to generalize. Our study utilized only fixed and demographic variables that can be validated in larger, prospective studies to define infant risk for PH during the neonatal course. In this way, clinicians could target serial echocardiography to higher-risk infants, and potentially impact postnatal PH surveillance and management in a timely manner.

## Conclusions

The presence of an atrial septal defect and intrauterine growth restriction strongly increased the odds of pulmonary hypertension that persists at or near-term in very low birthweight premature infants. Premature infants in referral neonatal intensive care units with these risk factors should potentially have PH evaluations performed earlier or more frequently in their hospital course.

## Abbreviations

BPD: Bronchopulmonary dysplasia
late PH: Late pulmonary hypertension
